# Supplementary figures and images for: Ubiquitination drives COPI priming and Golgi SNARE localization
Source: eLife. 2022 Jul 29;11:e80911. doi: 10.7554/eLife.80911 (PMC9374436; doi:10.7554/eLife.80911)

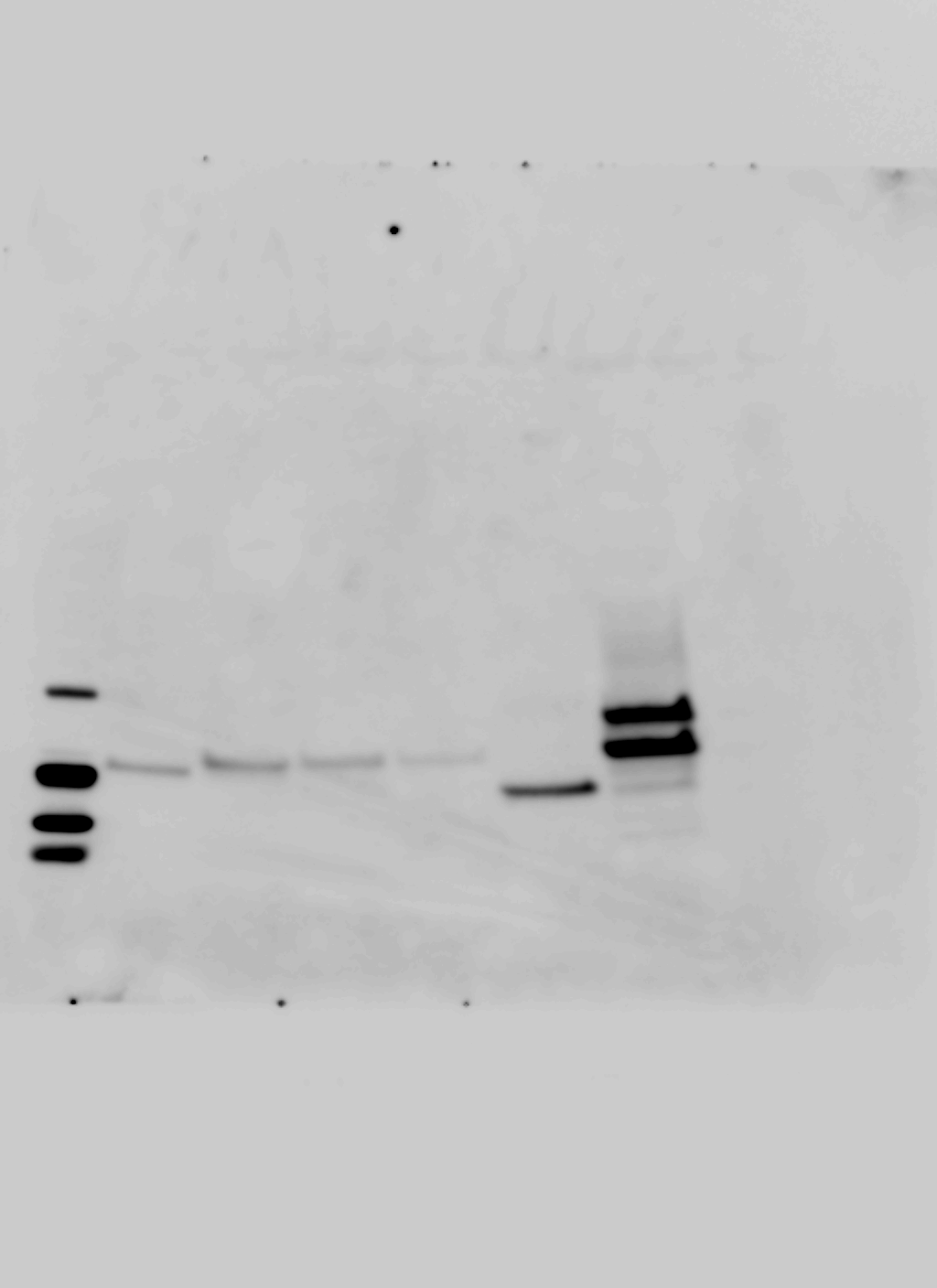

Supplement: Source data 1. [file elife-80911-data1.zip › Figure 3 Source Data - Blot 1.png]

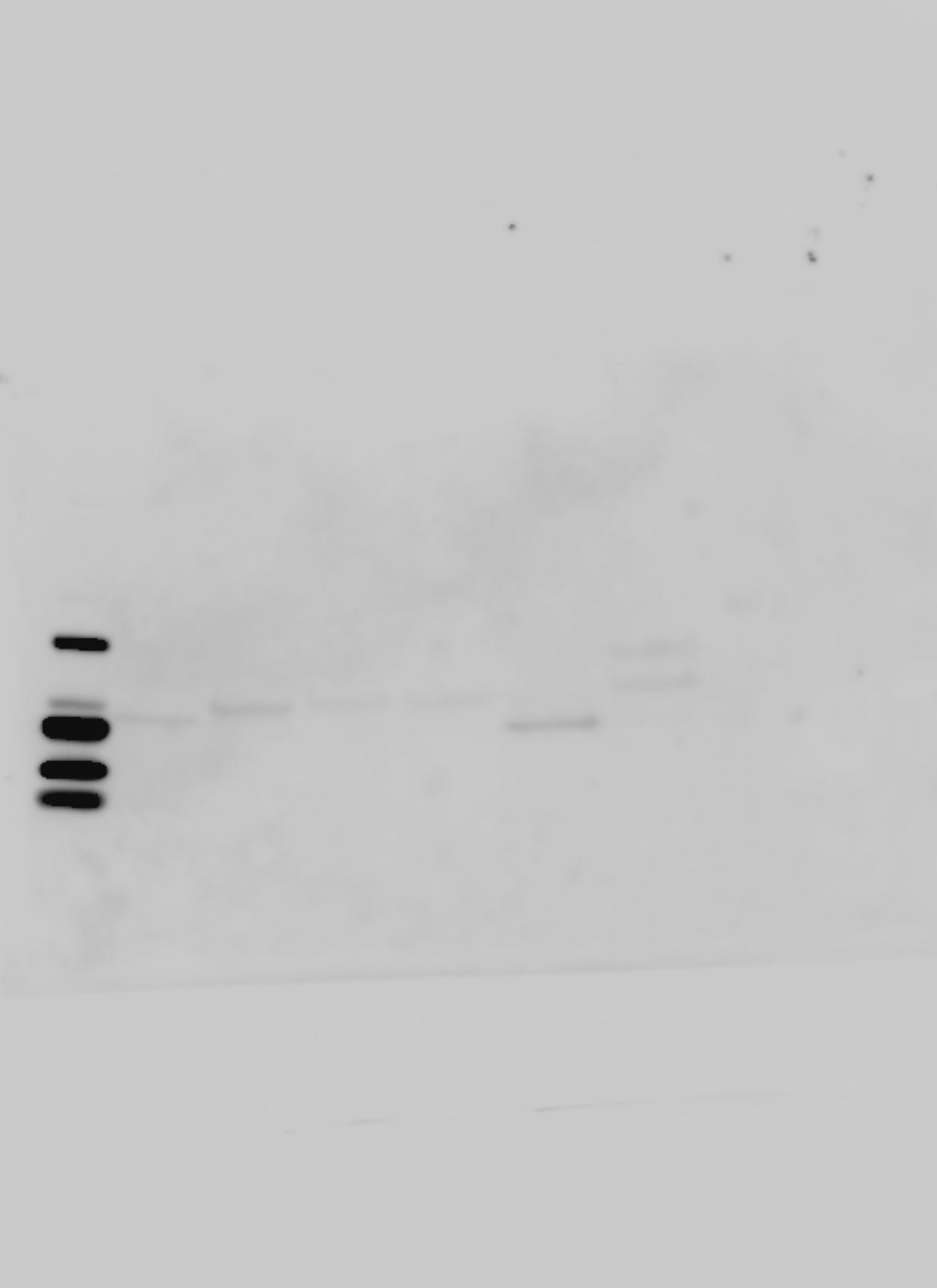

Supplement: Source data 1. [file elife-80911-data1.zip › Figure 3 Source Data - Blot 2.png]

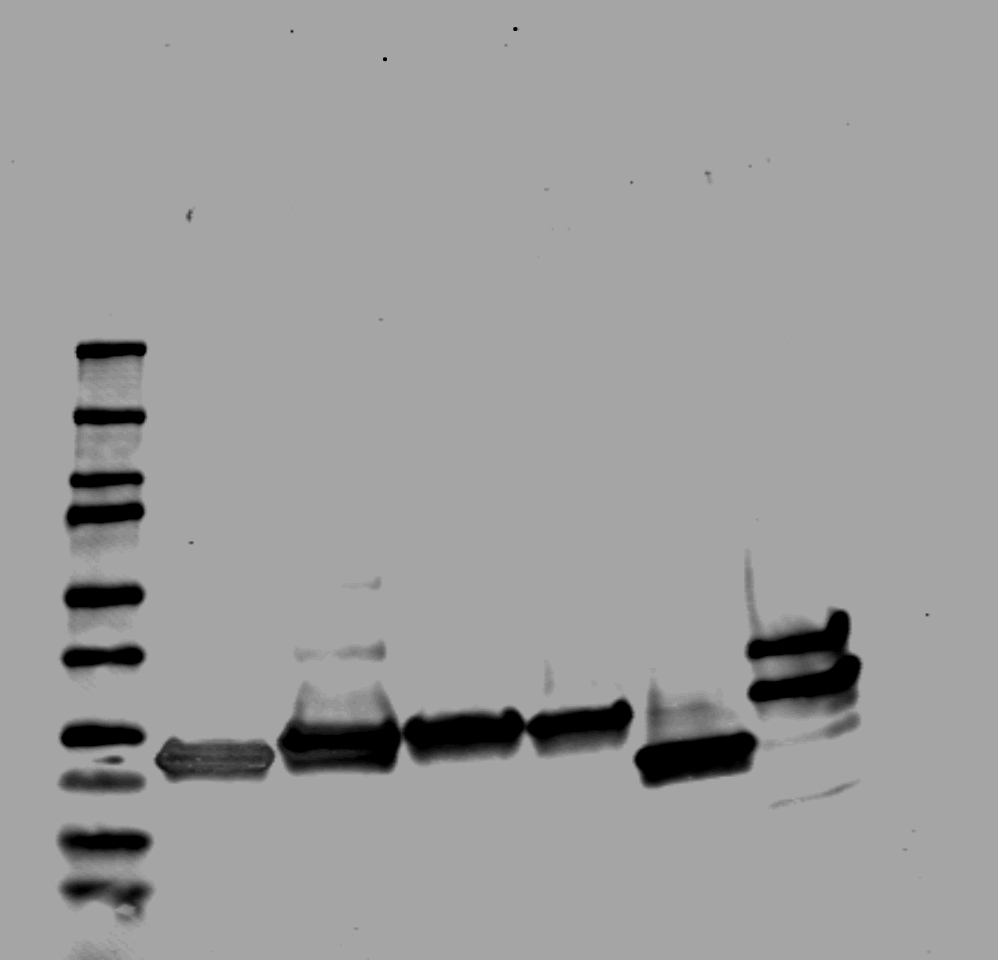

Supplement: Source data 1. [file elife-80911-data1.zip › Figure 3 Source Data - Blot 3.png]

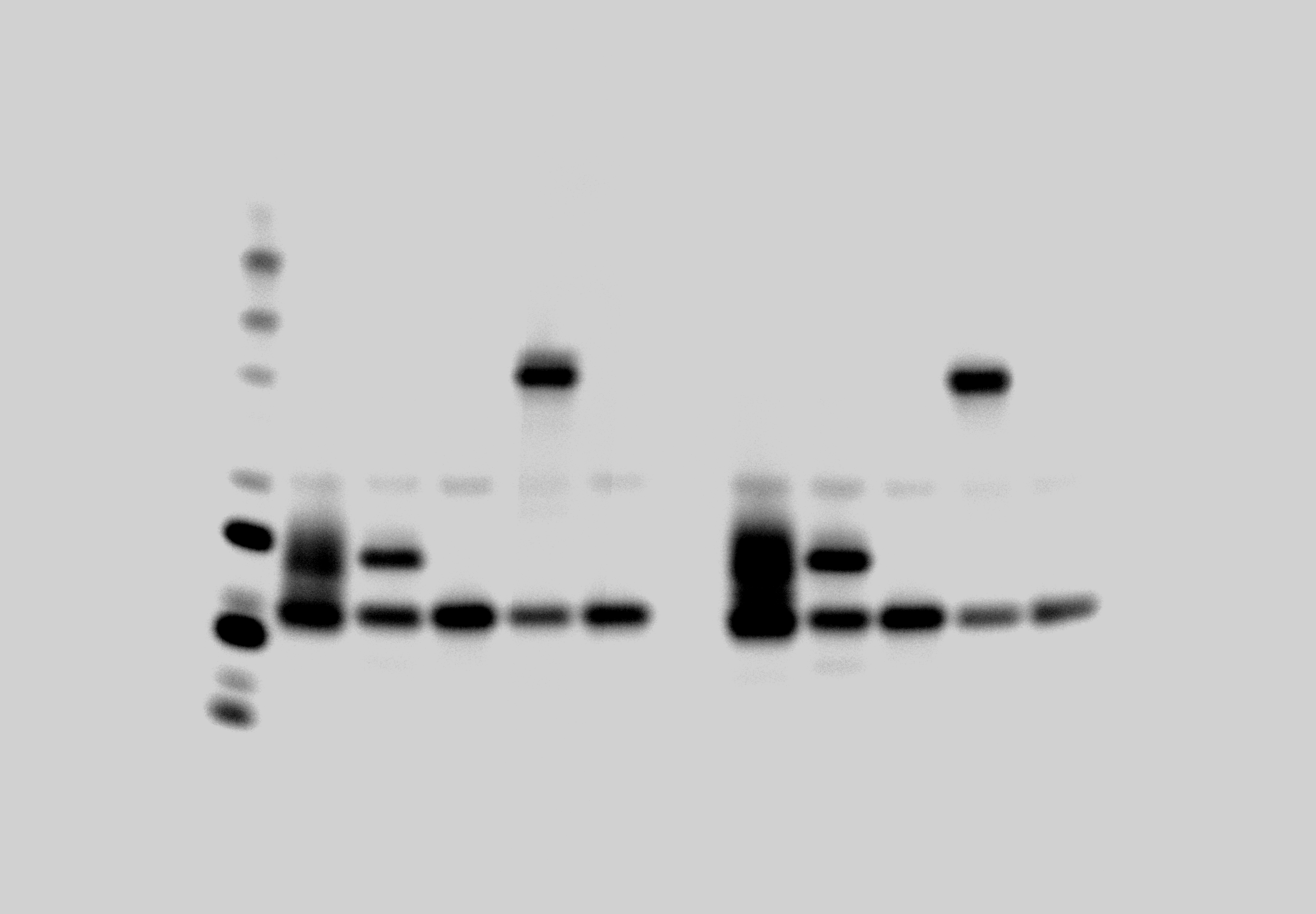

Supplement: Source data 1. [file elife-80911-data1.zip › Figure 5 Source Data - Blot 1.png]

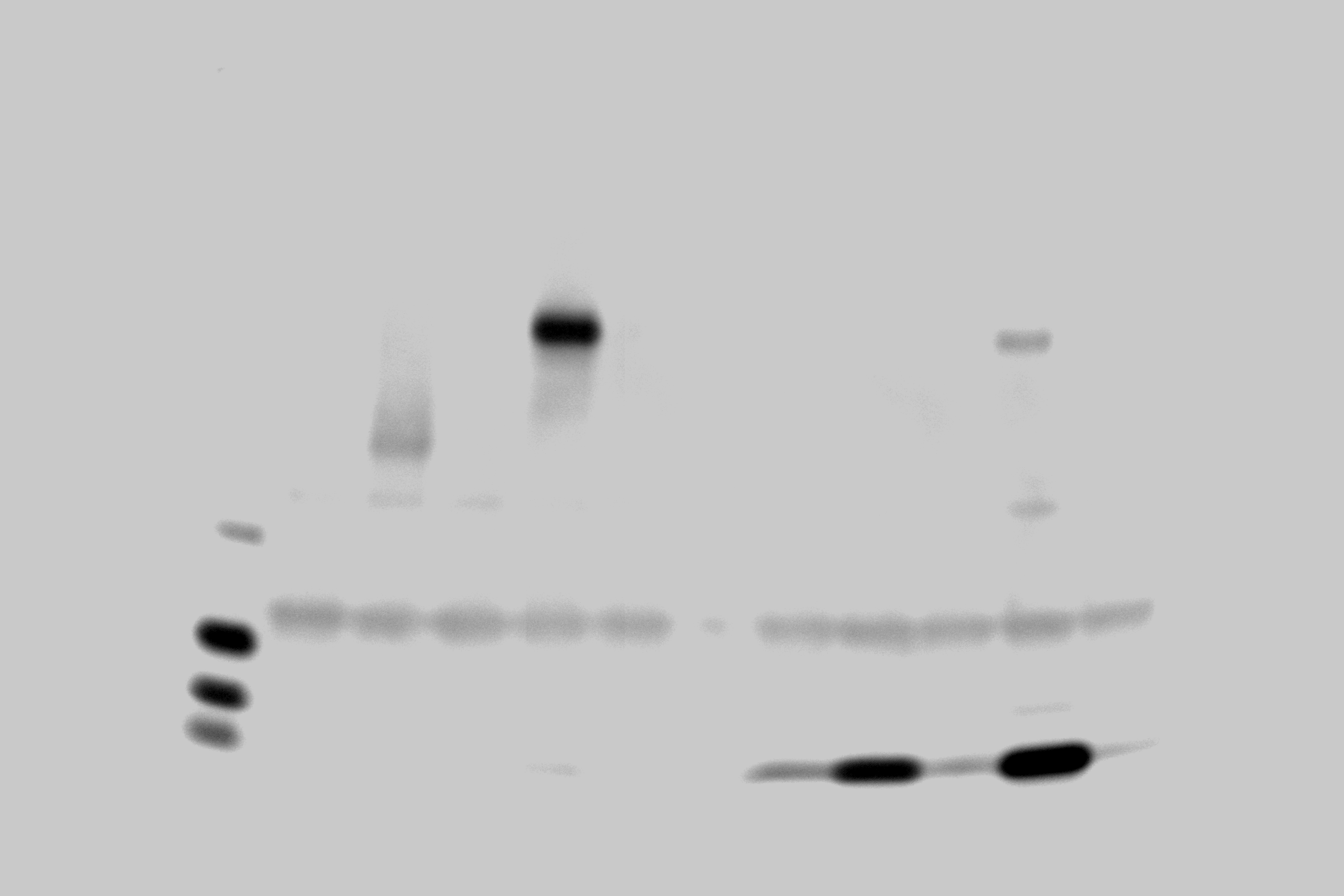

Supplement: Source data 1. [file elife-80911-data1.zip › Figure 5 Source Data - Blot 2.png]

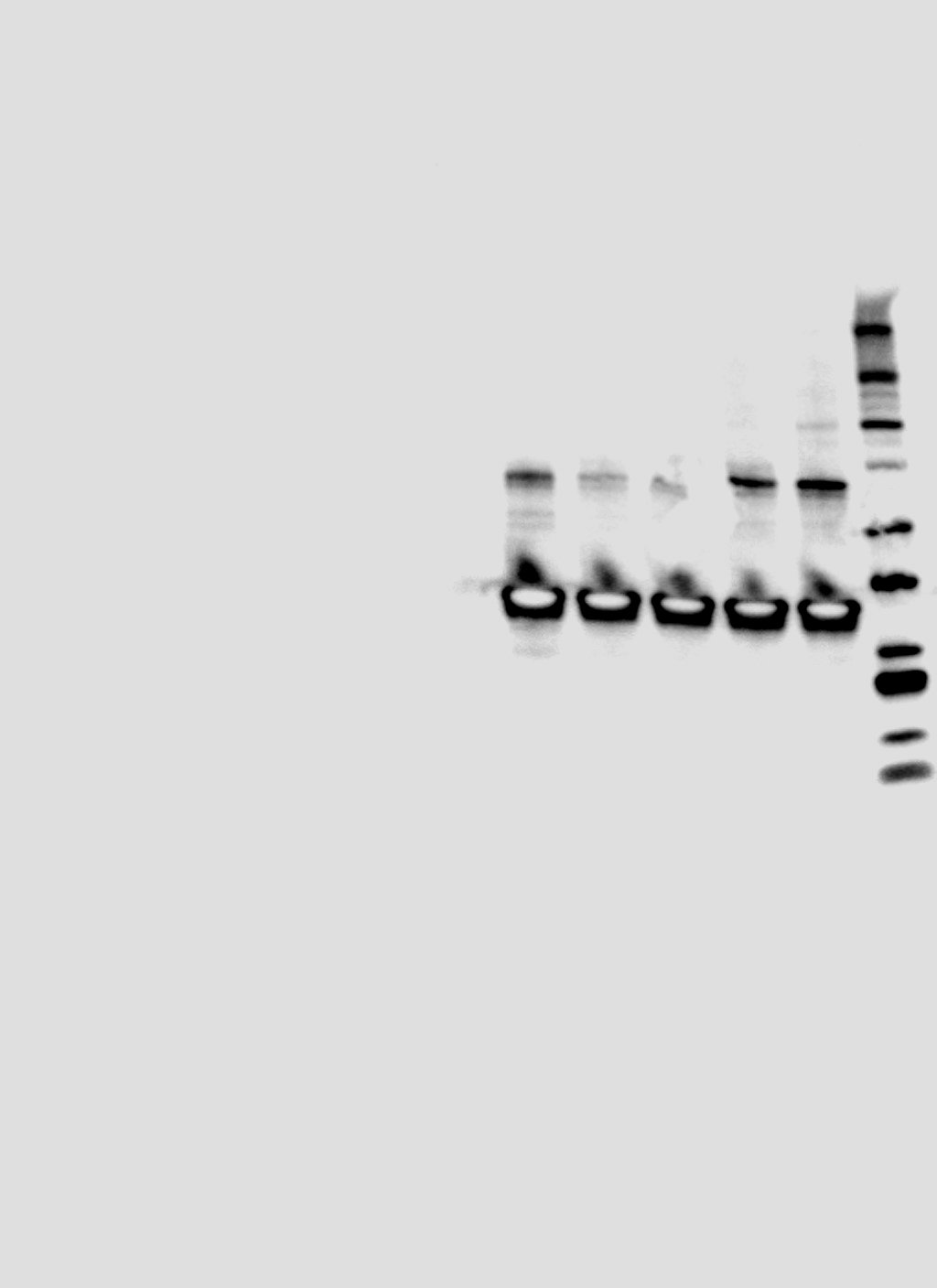

Supplement: Source data 1. [file elife-80911-data1.zip › Figure 5 Source Data - Blot 3.jpg]

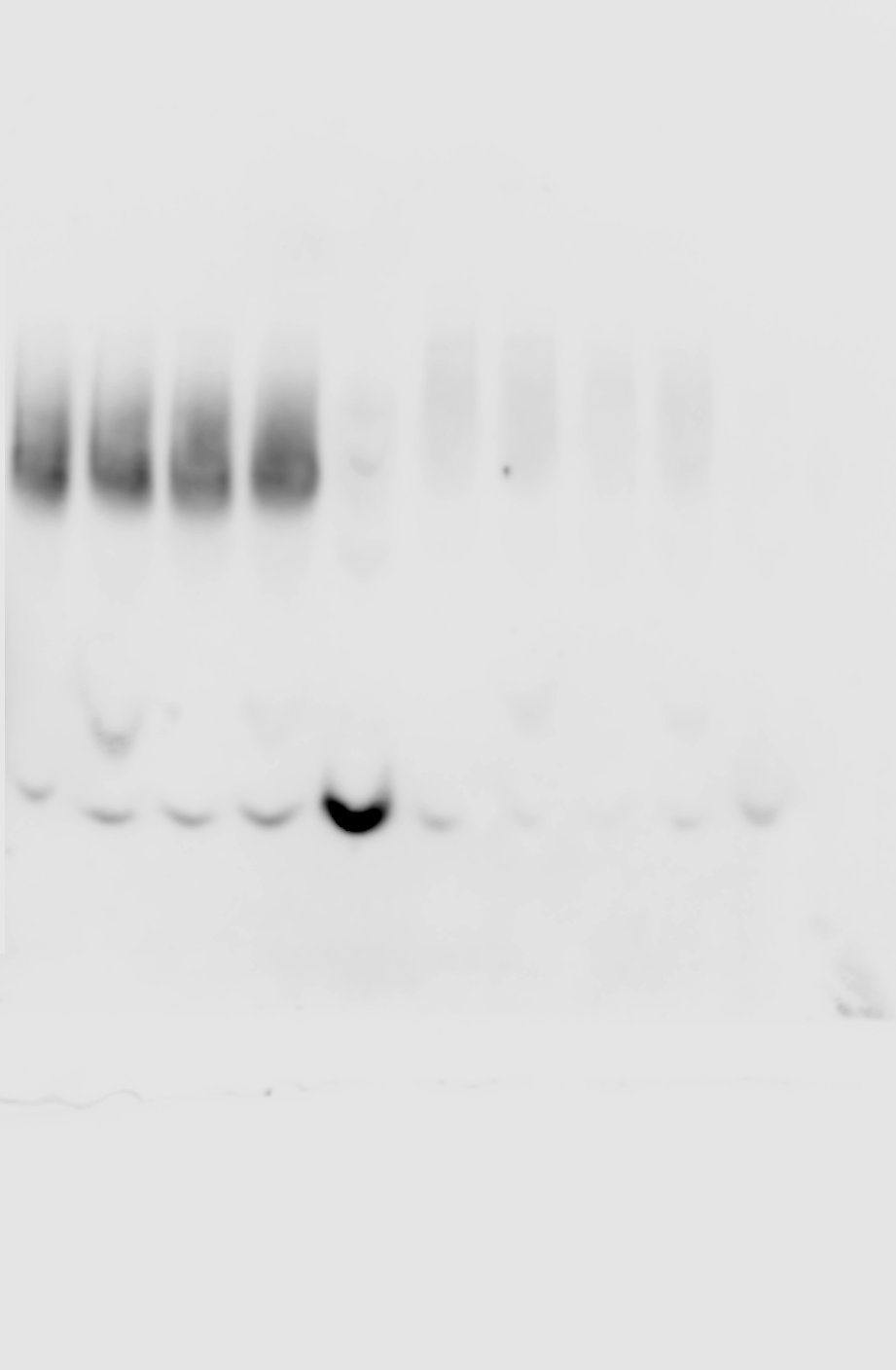

Supplement: Source data 1. [file elife-80911-data1.zip › Figure 5 Source Data - Blot 4.png]

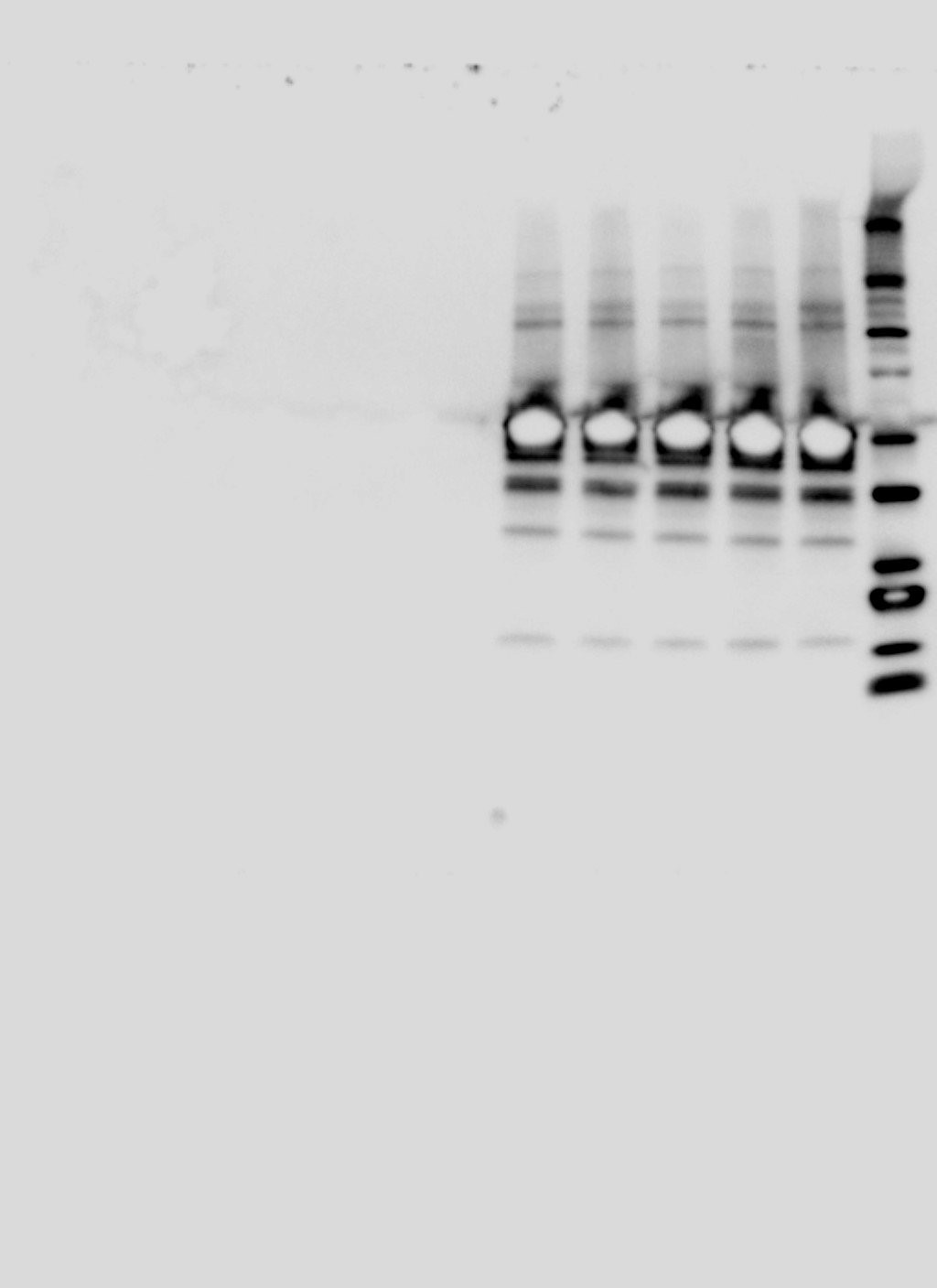

Supplement: Source data 1. [file elife-80911-data1.zip › Figure 5 Source Data - Blot 5.jpg]

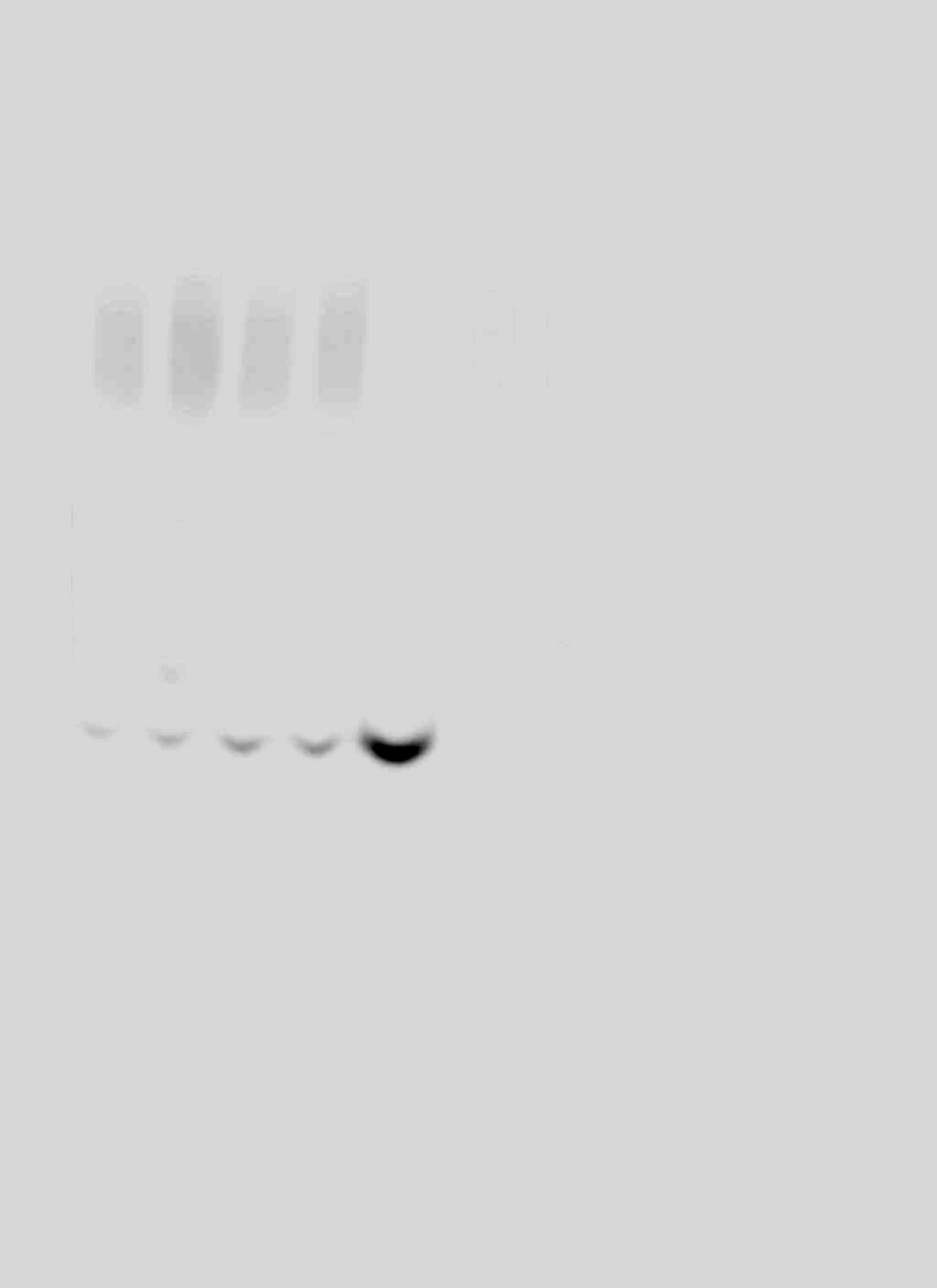

Supplement: Source data 1. [file elife-80911-data1.zip › Figure 5 Source Data - Blot 6.jpg]

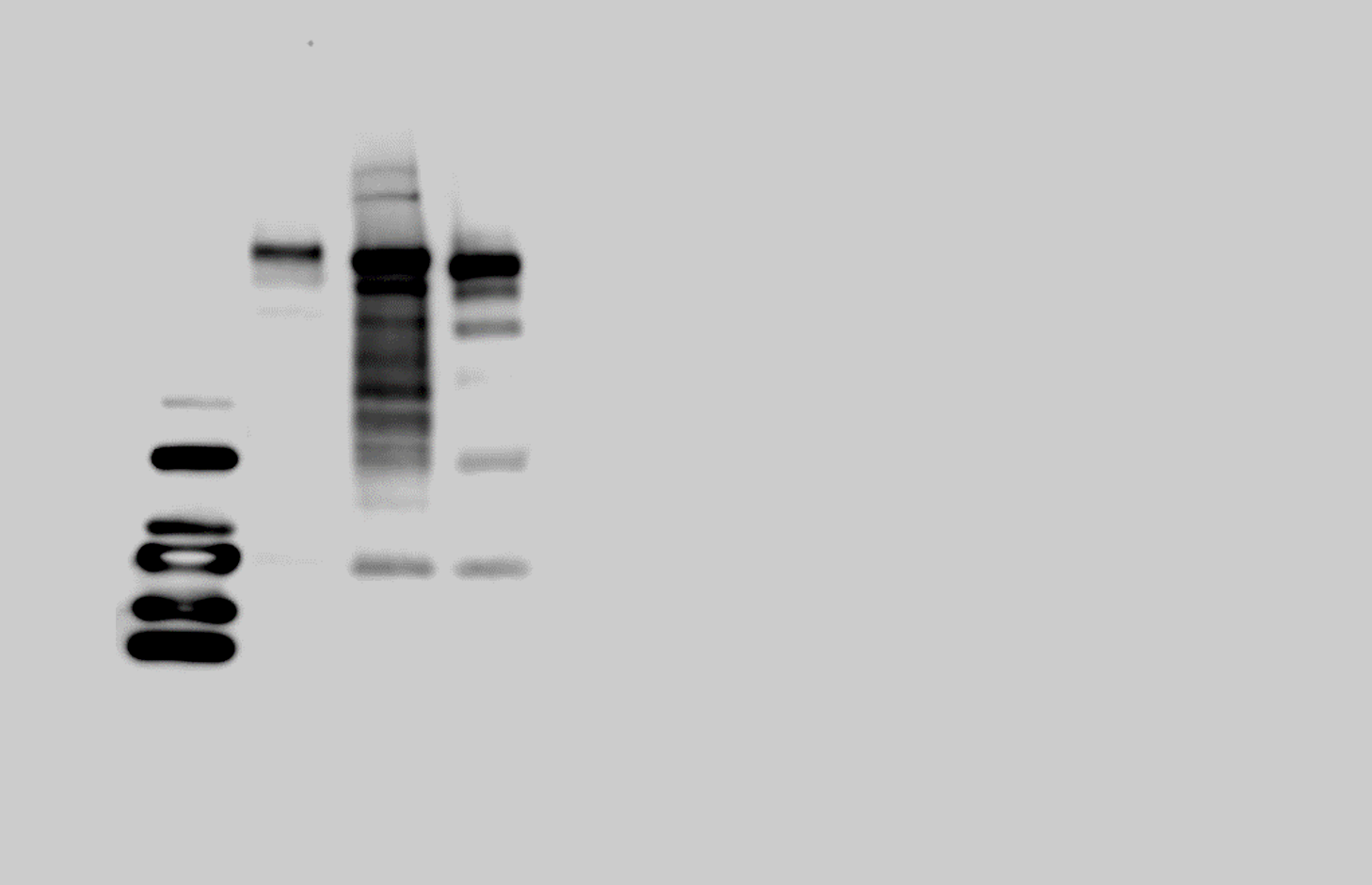

Supplement: Source data 1. [file elife-80911-data1.zip › Figure 6 Source Data - Blot 1.png]

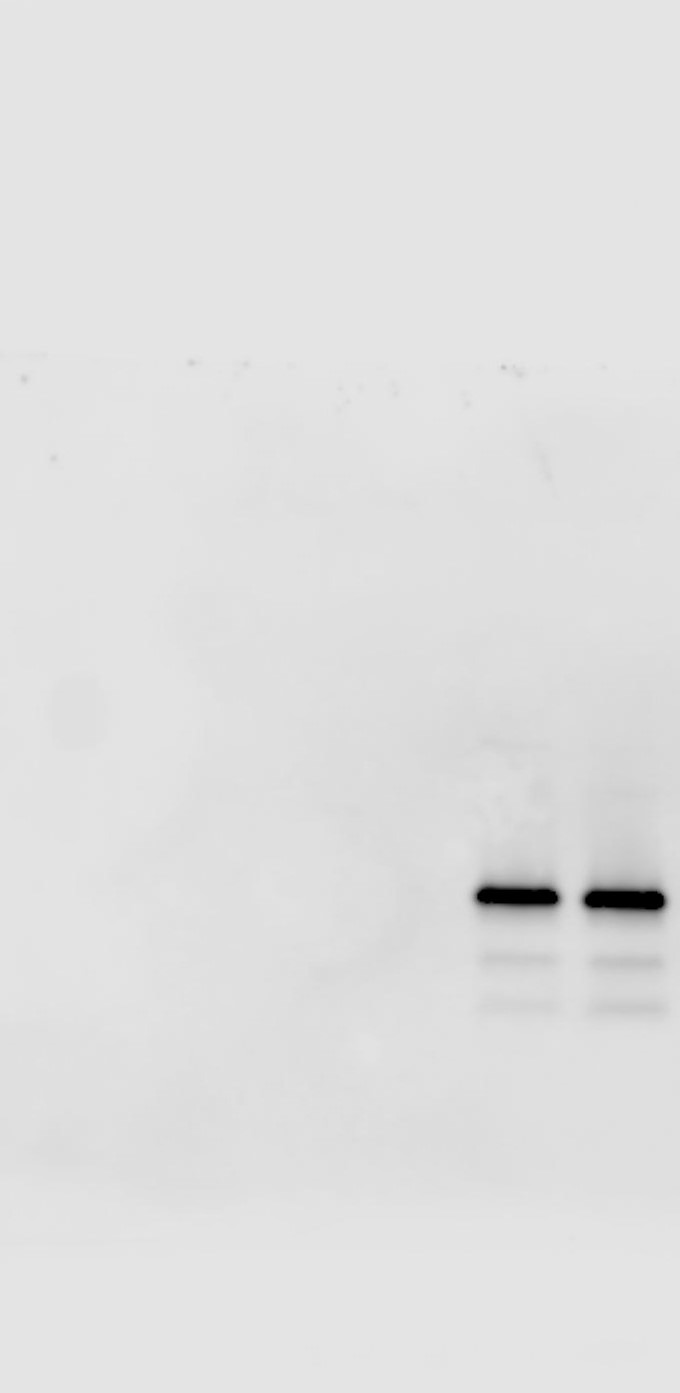

Supplement: Source data 1. [file elife-80911-data1.zip › Figure 6 Source Data - Blot 2.jpg]

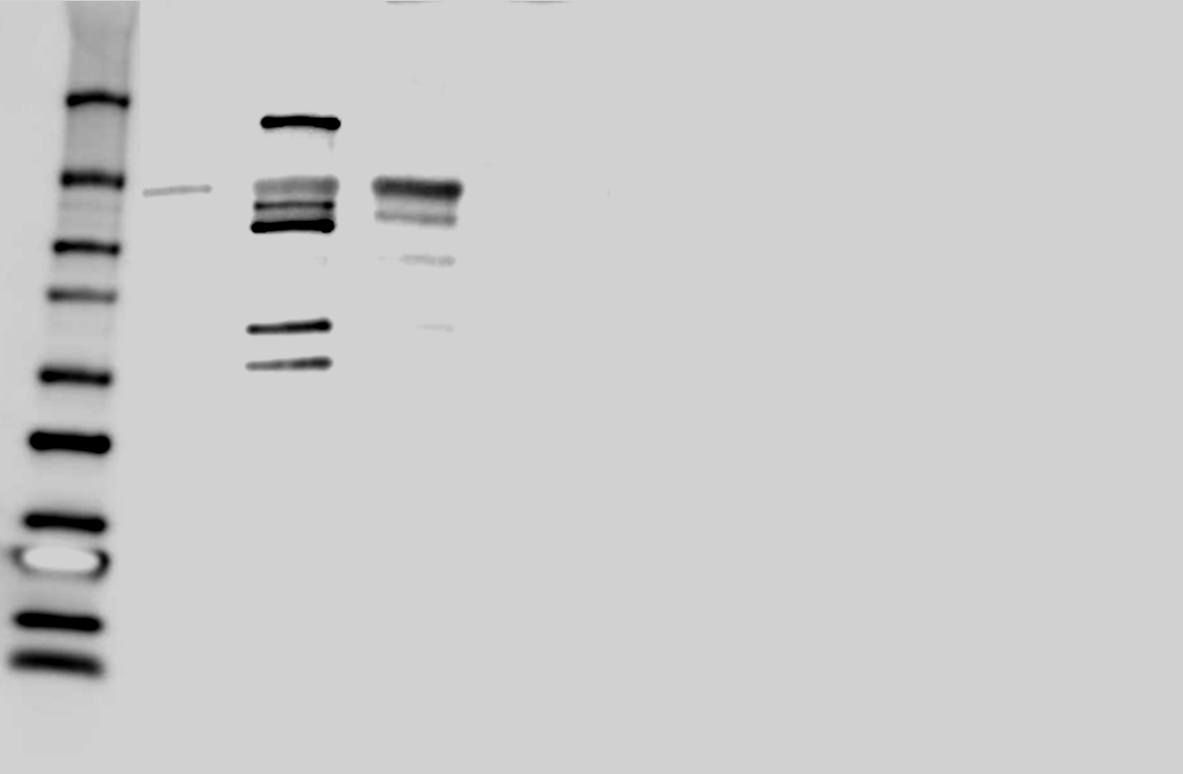

Supplement: Source data 1. [file elife-80911-data1.zip › Figure 6 Source Data - Blot 3.jpg]

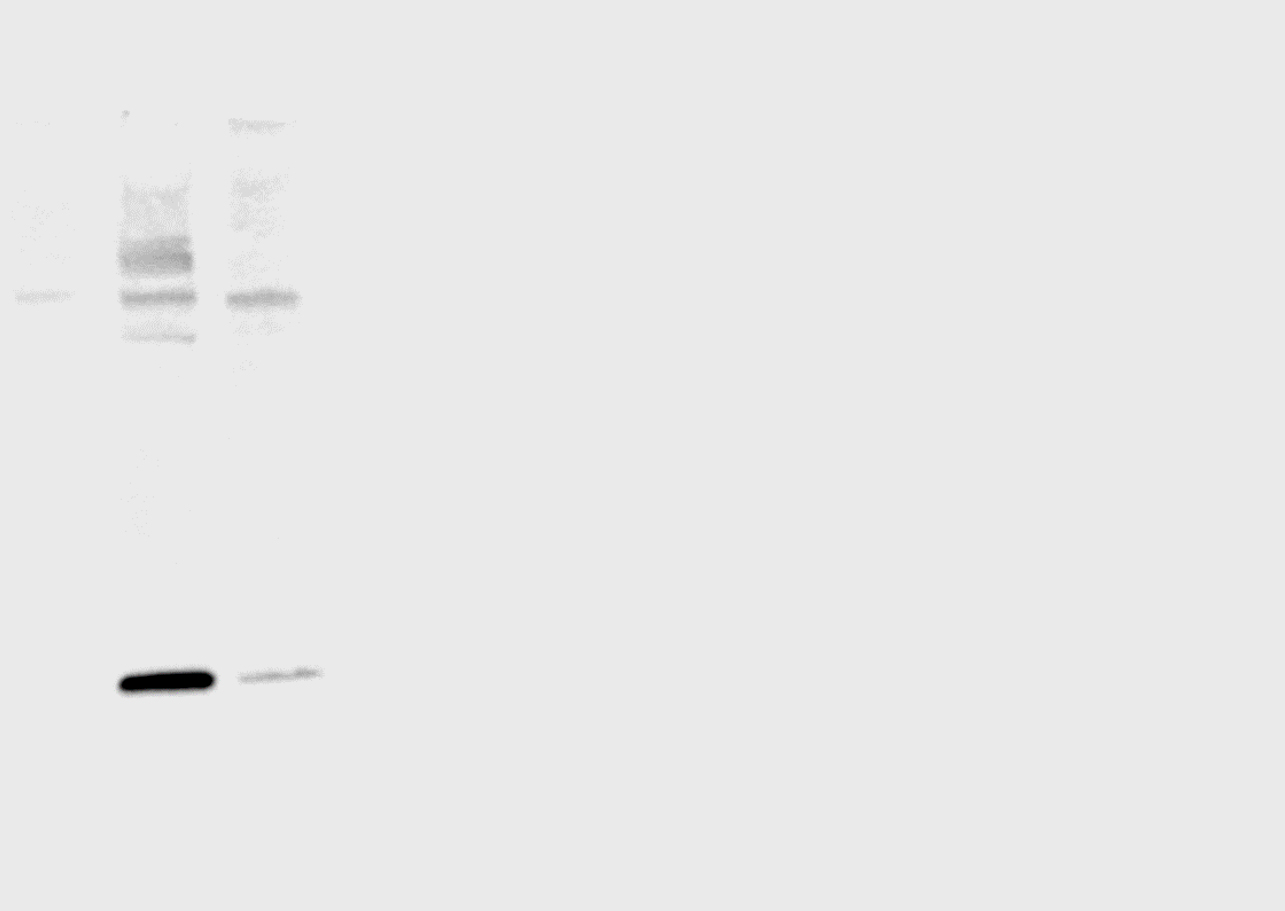

Supplement: Source data 1. [file elife-80911-data1.zip › Figure 6 Source Data - Blot 4.jpg]

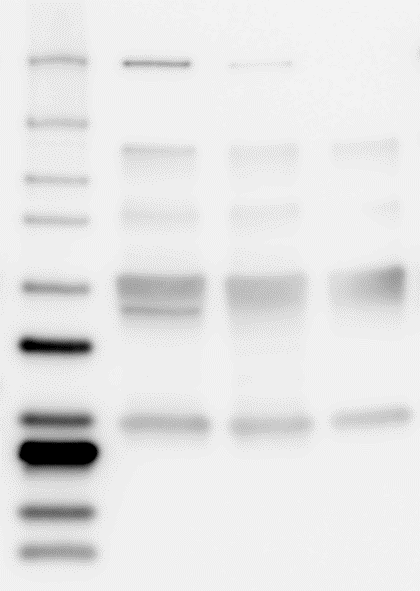

Supplement: Source data 1. [file elife-80911-data1.zip › Figure 7 Source Data - Blot 1.png]

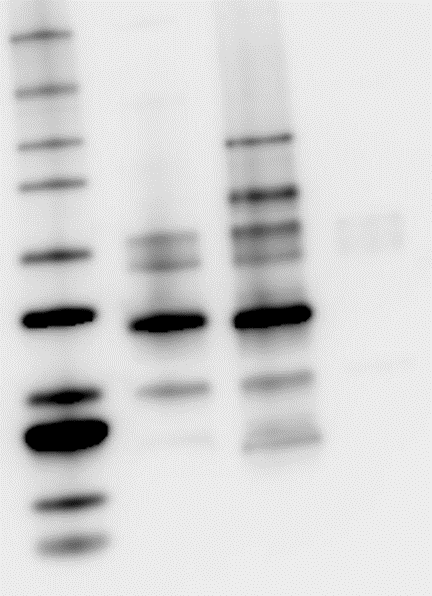

Supplement: Source data 1. [file elife-80911-data1.zip › Figure 7 Source Data - Blot 2.png]

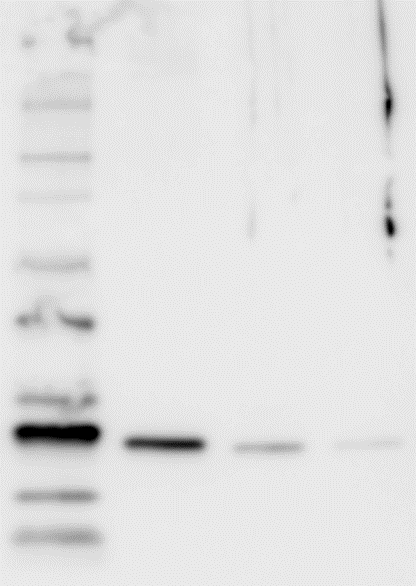

Supplement: Source data 1. [file elife-80911-data1.zip › Figure 7 Source Data - Blot 3.png]

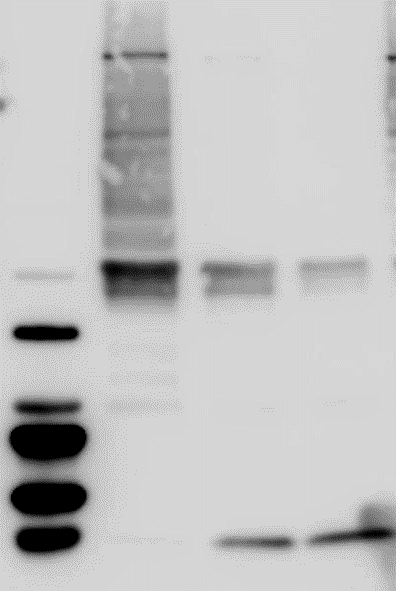

Supplement: Source data 1. [file elife-80911-data1.zip › Figure 7 Source Data - Blot 4.png]

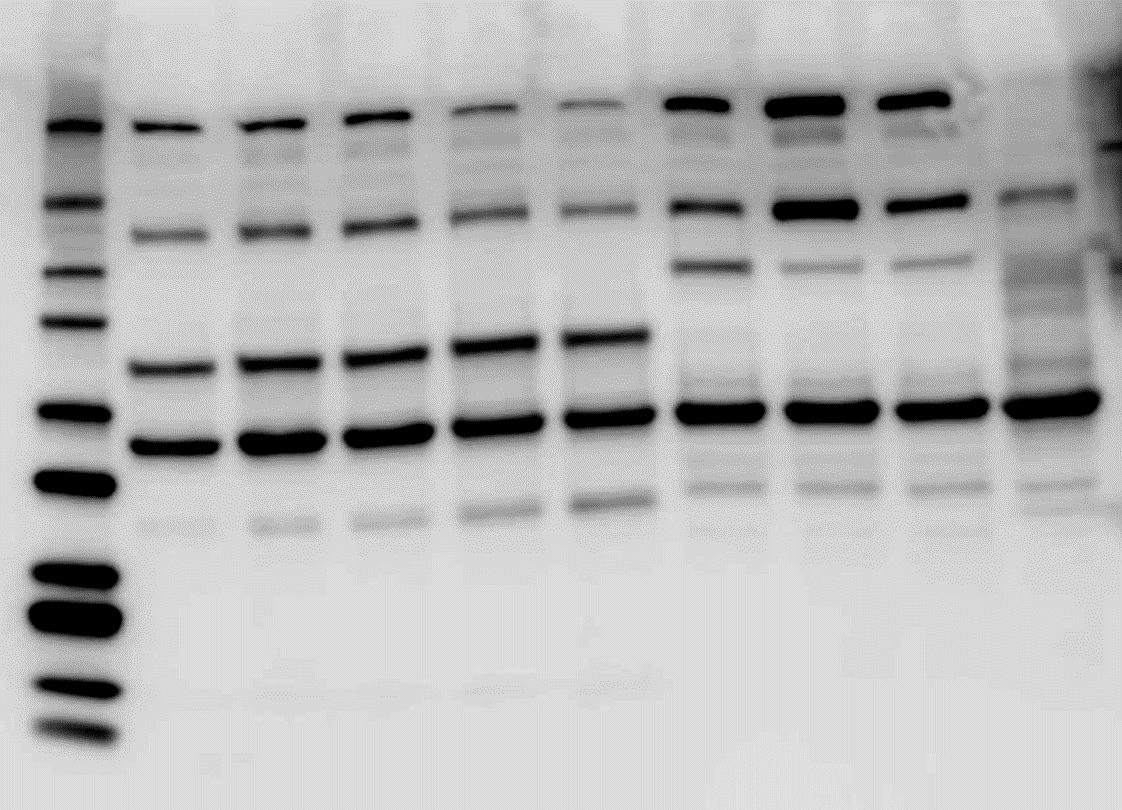

Supplement: Source data 1. [file elife-80911-data1.zip › Figure 7 Source Data - Blot 5.jpg]
